# Case Report: Fulminant shock due to PVL-positive *Staphylococcus aureus* in an adolescent—superantigen-negative sepsis with toxic shock-like features

**DOI:** 10.3389/fmed.2026.1729510

**Published:** 2026-01-16

**Authors:** Rachid Attou, Sohaïb Mansour, Evelyne Maillart, Leonel Barreto Gutierrez, Ayoub Jaafari

**Affiliations:** 1 Intensive Care Department, CHU Brugmann, Brussel, Belgium; 2 Infectious Disease Department, CHU Brugmann, Brussel, Belgium

**Keywords:** mixed shock, Panton-Valentine leukocidin, pulmonary embolism, superantigen-negative, toxin suppression, VA-ECMO

## Abstract

**Background:**

Panton-Valentine leukocidin (PVL)-positive *Staphylococcus aureus* strains are associated with severe necrotising infections and may be linked to fulminant systemic inflammatory presentations. We report an exceptionally fulminant and rapidly fatal case of PVL-positive *S. aureus* sepsis with toxic shock-like features and early deterioration despite escalation to veno-arterial extracorporeal membrane oxygenation (VA-ECMO).

**Case presentation:**

A previously healthy 16-year-old boy presented with chest pain, dyspnoea and circulatory collapse. Initial evaluation demonstrated massive bilateral pulmonary embolism, severe biventricular systolic dysfunction, acute kidney injury and marked systemic inflammation. He received prompt haemodynamic support, systemic thrombolysis for high-risk pulmonary embolism and empirical broad-spectrum antibiotics. Echocardiography confirmed profound myocardial dysfunction and VA-ECMO was instituted as salvage support. *S. aureus* grew from respiratory samples and blood cultures, with susceptibility consistent with meticillin-susceptible *S. aureus*. Virulence gene profiling by multiplex PCR detected *lukS-PV and LukF-PV*, while *tst*, *eta* and *etb* were not detected. Antimicrobial therapy was shifted to include antitoxin agents (clindamycin and linezolid). Intravenous immunoglobulin was not administered. Despite maximal supportive care, he developed refractory multi-organ failure and died within 24 h of ICU admission.

**Conclusion:**

PVL-positive *S. aureus* can, albeit rarely, be associated with an extreme toxic shock-like phenotype even when classical toxin genes are not detected. This case highlights the diagnostic and management challenges posed by atypical presentations and mixed shock physiology, and underscores the need for early recognition and rapid escalation of supportive care.

## 1 Introduction

Toxic shock syndrome (TSS) is a life-threatening, toxin-mediated systemic illness most often associated with toxigenic *Staphylococcus aureus* (*S. aureus*) and group A *Streptococcus* (1, 2). These organisms can produce superantigens such as toxic shock syndrome toxin 1 and staphylococcal enterotoxins, which bypass conventional antigen processing and trigger abrupt polyclonal T-cell activation, leading to a systemic cytokine surge (1, 3). The ensuing inflammatory cascade drives endothelial activation, vasoplegia, capillary leak, coagulopathy and myocardial depression, culminating in shock and multi-organ dysfunction (1, 3). Although originally recognized in menstrual disease, non-menstrual TSS occurs across all ages and may present without classical features such as fever or rash, which can delay recognition, particularly in children and adolescents (2, 4). We report a previously healthy 16-year-old with a fulminant toxic shock-like presentation due to PVL-positive *S. aureus*, complicated by severe biventricular dysfunction and massive pulmonary embolism, resulting in mixed shock, defined as the coexistence of distributive (vasoplegic), cardiogenic and obstructive components.

## 2 Case presentation

A previously healthy 16-year-old male presented to the emergency department with oppressive chest pain and progressive dyspnoea. On arrival, he was hypotensive (75/36 mmHg), tachycardic (120 bpm), and tachypneic (30 breaths/min) with cold extremities and delayed capillary refill. He was afebrile (36.1 °C). Pulmonary auscultation revealed bibasilar crackles. Initial arterial blood gas (ABG) analysis revealed compensated metabolic acidosis (pH 7.40, pCO₂ 32 mmHg, pO₂ 84 mmHg on room air) and elevated serum lactate (4.6 mmol/L). Laboratory investigations demonstrated marked systemic inflammation and early multi-organ dysfunction, including CRP 286.0 mg/L, severe thrombocytopenia of 42,000/μL, coagulopathy with D-dimers 24,901 ng/mL, elevated aspartate aminotransferase (ASAT/GOT) and lactate dehydrogenase (LDH), acute kidney injury with creatinine 3.16 mg/dL, myocardial injury with troponin 101.2 ng/L, as summarized in Table 1.

**Table 1:** Laboratory findings of the patient.

Analysis	Result	Units	Reference range
Hemoglobin	9.10	g/dL	13.0–18.0
RBC	3.20	×10^6^/μL	4.40–5.90
Hematocrit	26.10	%	40.0–53.0
MCV	82.0	fL	80–100
Platelets	42.0	×10^3^/μL	150–440
WBC	2.25	×10^3^/μL	3.50–11.00
NRBC /100 WBC	30.0	%	<1
Neutrophils (abs)	1.24	×10^3^/μL	1.50–6.70
Lymphocytes (abs)	1.29	×10^3^/μL	1.20–3.50
Monocytes (abs)	0.20	×10^3^/μL	0.10–1.00
Eosinophils (abs)	0.02	×10^3^/μL	0.10–0.50
Reticulocytes (%)	0.86	%	1.06–2.63
CRP	286.0	mg/L	<5.0
PT	20.7	s	9.9–11.8
APTT ratio	2.52	s	0.80–1.20
Fibrinogen	122	mg/dL	150–400
D-dimers	24,901	ng/mL	<500
Sodium	148	mmol/L	136–145
Potassium	7.0	mmol/L	3.4–4.7
Bicarbonate	15	mmol/L	23–29
Albumin	18	g/L	32–45
Urea	52.3	mg/dL	15.0–42.8
Creatinine	3.16	mg/dL	0.70–1.20
eGFR (CKD-EPI)	44	mL/min/1.73m^2^	>60
ALT (GPT)	507	U/L	<18
LDH	4,159	U/L	<240
Bilirubin (total)	2.3	mg/dL	<1.2
Creatine kinase (CK)	72,941	U/L	39–308
Troponin T	101.2	ng/L	<14.0
Glucose	68	mg/dL	70–100

**Figure 1:**
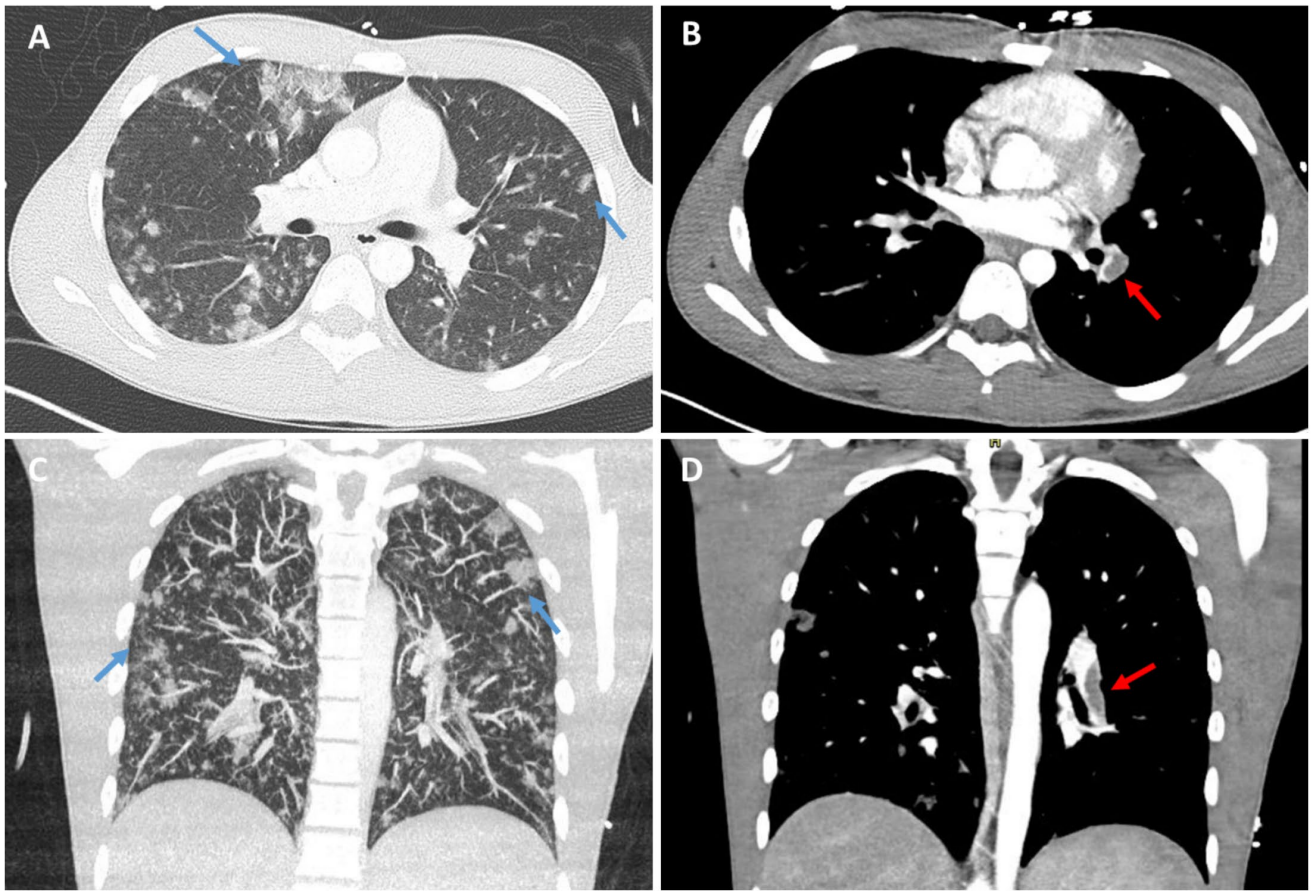
Chest CT findings in a 16-year-old male with PVL-positive *Staphylococcus aureus* sepsis. **(A,C)** Axial **(A)** and coronal **(C)** lung window images show multifocal bilateral peripheral ground-glass opacities and consolidations (blue arrows), suggestive of acute inflammatory or infectious process, compatible with septic pulmonary emboli or hemorrhagic pneumonia. **(B,D)** Axial **(B)** and coronal **(D)** contrast-enhanced mediastinal window images demonstrate filling defects within the right pulmonary artery (red arrows), suggestive of acute pulmonary embolism.

Urgent CT pulmonary angiography confirmed the presence of bilateral pulmonary embolism, with proximal occlusion of the left main and lobar pulmonary arteries, and distal thrombi in the right-sided branches (Figures 1B,D). Additionally, there were bilateral ground-glass opacities and peripheral consolidations consistent with an inflammatory or infective process (Figures 1A,C). Given haemodynamic instability and imaging consistent with high-risk pulmonary embolism, systemic thrombolysis was administered in accordance with guideline-based management. He subsequently required endotracheal intubation and vasopressor support with norepinephrine. Empirical broad-spectrum antimicrobial therapy was initiated immediately with amikacin and amoxicillin-clavulanate (Table 2).

**Table 2:** Comparative summary of published pediatric and adolescent/young adult reports of PVL-positive *Staphylococcus aureus* infection associated with shock.

Case	Age	Toxin profile	PE Present	LVEF	Antitoxin therapy	ECMO	Outcome
Mushtaq et al. (22)	14 y	PVL+, TSST-1+, SEC+	No	Unknown	Not specified	No	Death
Li et al. (23)	Teen	PVL+, TSST-1+	No	Unknown	Not specified	No	Death
Hayakawa et al. (24)	20 & 61 y	PVL+, TSST-1+	No	Unknown	Yes (1 case)	No	1 death / 1 survival
Chi et al. (25)	Pediatric cohort	PVL+, seb+	No	Unknown	Not specified	No	High mortality
Cuddihy et al. (26)	Teen	PVL + only	No	Unknown	Yes + ECMO	Yes	Survival

Compared with prior cases, the present case was notable for an exceptionally fulminant course and for PVL positivity with no classical superantigen or exfoliative toxin genes detected on multiplex PCR. In most published reports, PVL was accompanied by additional toxin genes, including *tst* encoding TSST-1 and/or enterotoxins. Use of antitoxin antibiotics (e.g., clindamycin, linezolid) and extracorporeal support varied across cases. Overall survival was uncommon, underscoring the potential severity of toxin-associated *S. aureus* shock and the need for early recognition and aggressive supportive management.

Despite a brief haemodynamic response, the patient progressed to refractory circulatory failure. Bedside echocardiography revealed severe biventricular systolic dysfunction with a left ventricular ejection fraction (LVEF) below 20%. Dobutamine was commenced for inotropic support; however, ongoing deterioration prompted initiation of veno-arterial extracorporeal membrane oxygenation (VA-ECMO).

A tracheobronchial aspirate yielded abundant growth of *S. aureus*. Antimicrobial susceptibility testing was performed using the VITEK 2 system (bioMérieux) and interpreted according to EUCAST criteria. The isolate was consistent with meticillin susceptible *S. aureus* (MSSA), with susceptibility to oxacillin, clindamycin, linezolid, vancomycin, rifampicin and trimethoprim sulfamethoxazole; ciprofloxacin was reported as intermediate. Blood cultures also grew *S. aureus, and were* obtained at presentation, prior to initiation of empirical antibiotics. Preliminary Gram-positive cocci were reported within 8 h, and full susceptibility results became available approximately 18 h later, shortly before escalation to clindamycin and linezolid. Virulence gene profiling was performed on the blood culture isolate using a multiplex PCR assay, which detected genes encoding Panton-Valentine leukocidin (*lukS-PV/lukF-PV*), whereas genes encoding toxic shock syndrome toxin 1 (*tst*) and exfoliative toxins A (*eta*) and B (*etb*) were not detected. In light of a suspected toxin-mediated syndrome, antimicrobial therapy was shifted to include clindamycin and linezolid, aiming to suppress exotoxin production via inhibition of bacterial protein synthesis. Adjunctive intravenous immunoglobulin was not administered.

**Figure 2:**
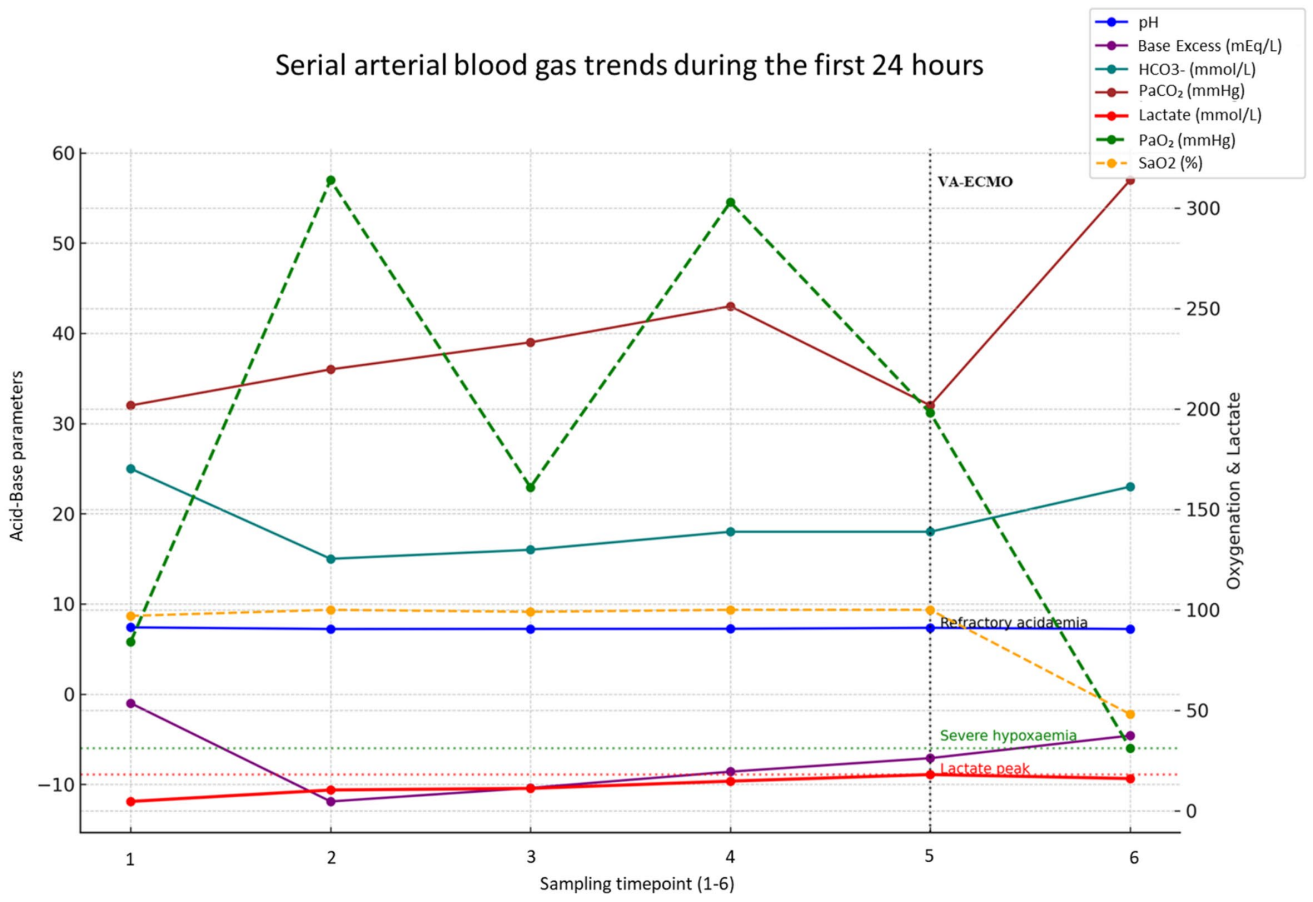
Serial arterial blood gas trends during the first 24 h of ICU admission. Arterial blood gas samples were obtained during the first 24 h (sample 1 at presentation and sample 6 immediately prior to death). Acid–base variables are shown on the left *y*-axis (pH, base excess, bicarbonate HCO_3_^−^, PaCO_2_, lactate, PaCO_2_, and SaO_2_). Oxygenation indices and lactate are shown on the right *y*-axis (PaO_2_, SaO_2_, and lactate). Progressive acidaemia (final pH 7.22) and rising lactate (peak 18 mmol/L) were accompanied by worsening hypoxaemia (final PaO_2_ 31 mmHg). The timing of VA-ECMO initiation is indicated at sample 5. The pattern underscores the rapid deterioration associated with toxin-mediated shock and severe cardiopulmonary compromise.

Serial ABG analysis demonstrated a rapid and profound deterioration in both metabolic and respiratory parameters, as depicted in Figure 2. Lactate levels rose steeply, peaking at 18 mmol/L, while progressive acidaemia culminated in a final arterial pH of 7.22. Oxygenation also worsened markedly, with a final PaO2 of 31 mmHg, consistent with escalating refractory shock, tissue hypoperfusion and irreversible multi-organ failure. The patient died within 24 h of admission to the intensive care unit.

## 3 Discussion

Microbiological characterization of the isolate identified a PVL-positive *S. aureus* isolate. PVL is a bicomponent pore-forming leukocidin composed of *LukS-PV* and *LukF-PV*, with marked tropism for human phagocytes. The S component, *LukS-PV*, binds specific host receptors, most notably C5aR1, also referred to as CD88, and C5aR2, also referred to as C5L2. This interaction is followed by assembly with *LukF-PV* and oligomerisation into a membrane pore, leading to rapid ionic dysregulation and phagocyte injury with subsequent lysis. The resulting release of neutrophil-derived mediators and danger-associated molecular patterns may further amplify inflammation (5, 6). Experimental human cell studies also show that PVL can activate the NLRP3 inflammasome in myeloid cells, with caspase 1 dependent processing and secretion of interleukin 1 beta and interleukin 18 (7). Beyond direct cytotoxicity, PVL-induced neutrophil lysis releases granule enzymes, reactive oxygen species and pro-inflammatory cytokines that can perpetuate endothelial injury, vasoplegia and capillary leak, key features overlapping with the systemic physiology of superantigen-mediated shock.

This provides biological plausibility for an exaggerated innate inflammatory response in severe PVL-positive infections, although the magnitude and clinical relevance *in vivo* are likely influenced by the broader virulence background of the strain and host susceptibility.

Importantly, the direct in-vivo contribution of PVL to virulence remains controversial. Early animal work suggested PVL could be sufficient to drive necrotising pneumonia (8). However, subsequent studies demonstrated that key phenotypes attributed to PVL in murine pneumonia models could be explained by an unintended *agr* locus mutation, rather than PVL itself, highlighting the complexity of translating experimental findings into causality (9). At the population level, a systematic review/meta-analysis supports an association of PVL with skin/soft-tissue infection and necrotising pneumonia, while relationships with invasive disease severity and mortality are inconsistent (10). Accordingly, this case cannot establish PVL causality; rather, it documents an unusually fulminant toxic shock-like phenotype caused by a PVL-positive *S. aureus* isolate.

In our patient, the abrupt haemodynamic collapse with rapid progression to multi-organ dysfunction is consistent with a toxin-associated hyper-inflammatory escalation. PVL positivity provides a biologically plausible contributor to this toxic shock-like phenotype, particularly given that classical toxin genes typically implicated in “classical” staphylococcal toxin-mediated syndromes were not detected. However, additional unmeasured bacterial virulence determinants and host susceptibility factors may also have contributed. Virulence gene profiling was performed on the blood culture isolate using a multiplex PCR assay with appropriate controls, which detected PVL genes while *tst, eta* and *etb* were not detected. These findings remain dependent on the targets included in the assay panel and therefore cannot fully exclude untested toxin genes.

From a clinical standpoint, this case highlights the rapid progression and lethality of toxin-mediated staphylococcal shock. In its classical form, TSS is driven by superantigens that bypass conventional antigen processing and trigger abrupt polyclonal T-cell activation, leading to a systemic cytokine surge, endothelial activation, vasoplegia, capillary leak, coagulopathy and multi-organ dysfunction (1, 3). Although fever and rash with subsequent desquamation are included in surveillance definitions, these features may be absent or delayed at presentation, which can hinder early recognition (2, 4) In our patient, the syndrome evolved within hours, with early cardiovascular collapse compatible with a mixed shock phenotype.

A striking feature was the combination of severe biventricular dysfunction and massive pulmonary embolism (PE), resulting in a mixed cardiogenic and obstructive haemodynamic profile. Sepsis is frequently complicated by sepsis-induced coagulopathy and, in severe cases, disseminated intravascular coagulation, reflecting bidirectional crosstalk between inflammation and coagulation. This dysregulated immune-thrombotic response promotes microvascular thrombosis, contributes to organ dysfunction, and is also associated with an increased risk of venous thromboembolism, including pulmonary embolism (11–13). In our patient, the combination of thrombocytopenia and markedly abnormal coagulation markers prompted early imaging, confirming massive bilateral PE. Management aligned with contemporary guidance for high-risk PE with haemodynamic instability, where systemic thrombolysis is recommended when not contraindicated (14).

Therapeutically, early initiation of antitoxin antibiotic therapy is biologically plausible and commonly recommended in suspected toxin-mediated syndromes. Protein synthesis inhibitors, notably clindamycin and linezolid, can reduce *de novo* exotoxin synthesis and modulate virulence factor expression *in vitro* and experimental models, although high-quality clinical outcome data in staphylococcal toxic shock syndrome remain limited (15, 16). With respect to PVL, antibiotic exposure can influence toxin expression. In particular, *β*-lactams may upregulate PVL production under certain conditions, whereas combinations with clindamycin, and to a lesser extent linezolid, have been shown to attenuate PVL induction in experimental settings (17, 18). In this context, the early shift to include clindamycin and linezolid as part of the antimicrobial strategy was rational as an attempt to suppress toxin production alongside haemodynamic support (15). Additionally, adjunctive intravenous immunoglobulin has been proposed as an adjunct in our patient with toxin-mediated shock; however, evidence remains limited and is mainly derived from streptococcal toxic shock syndrome, with insufficient robust data to support routine use in staphylococcal toxic shock-like presentations (19).

VA-ECMO was instituted as salvage support for refractory shock with severe myocardial dysfunction. Emerging observational data suggest that VA-ECMO may improve outcomes in carefully selected patients with sepsis-induced cardiogenic shock, a hypodynamic septic shock phenotype, whereas outcomes are poor when profound systolic dysfunction is absent or when irreversible multi-organ failure is already established (20, 21). In our patient, VA-ECMO was chosen because bedside echocardiography demonstrated severe biventricular systolic failure with ongoing haemodynamic collapse, indicating predominant circulatory failure rather than isolated respiratory insufficiency; VV-ECMO would not have provided circulatory support to address the cardiogenic component. In practice, the utility of VA-ECMO is limited by concomitant coagulopathy or disseminated intravascular coagulation, bleeding risk, and the rapid evolution of irreversible organ injury, constraints that were evident in our case despite early cannulation and maximal support.

Published reports indicate that PVL-positive *S. aureus* has been associated with toxic shock and necrotising pneumonia, most often in the setting of co-existing superantigen genes such as *tst* encoding TSST-1 and, in some cases, enterotoxins (22–24). In pediatric cohorts with staphylococcal toxic shock syndrome, carriage of *pvl* and *seb* appears more frequent among streptococcal toxic shock syndrome (STSS) isolates than among isolates from related toxin-mediated syndromes, supporting a potential link between these virulence determinants and severe clinical phenotypes (25). Fulminant toxic shock-like illness combining cardiogenic and obstructive shock, in the absence of detected classical toxin genes and without co-infection, appears exceptionally uncommon; the PVL-positive, superantigen-negative case reported by Cuddihy et al. is a rare example of survival following early antitoxin therapy and extracorporeal support (26). In contrast, our patient exhibited an even more fulminant course with rapid progression to refractory multi-organ failure within 24 h, and a distinctive mixed shock physiology driven by profound biventricular dysfunction combined with massive pulmonary embolism, a feature not reported in the available comparable PVL-only cases. This case therefore adds clinically important documentation of an extreme phenotype and supports the concept that PVL-positive, superantigen-negative isolates can be associated with devastating systemic disease, while explicitly acknowledging that association does not equate to proof of PVL-mediated causality.

This report has several limitations. First, its single-case design precludes any inference regarding causality or the frequency of similar presentations, and the observed association between PVL positivity and the toxic shock–like phenotype should not be interpreted as proof of PVL-mediated pathogenesis. Second, no post-mortem examination was performed; therefore, competing or contributory diagnoses (including the extent of pulmonary parenchymal involvement, myocardial pathology, and thrombotic burden) could not be fully characterized. Third, the portal of entry remained undetermined, limiting interpretation of the primary infectious focus and the chronology of toxin exposure.

From a microbiological perspective, virulence profiling relied on a targeted multiplex PCR panel; while appropriate controls were used, the results remain dependent on the assay’s predefined targets and cannot exclude untested toxin genes or broader genomic determinants that might influence virulence. In addition, circulating toxin levels and host inflammatory mediators were not quantified, and detailed immunological susceptibility testing was not undertaken. Finally, the coexistence of massive pulmonary embolism and severe myocardial dysfunction created complex mixed shock physiology, which may confound attribution of haemodynamic collapse to any single mechanism.

## 4 Conclusion

PVL-positive *Staphylococcus aureus* sepsis with no detected classical toxin genes typically implicated in toxin-mediated syndromes appears exceptionally uncommon in adolescents and may be associated with a fulminant toxic shock-like course. PVL offers biological plausibility for hyper-inflammation through phagocyte injury and inflammasome activation, although its contribution remains debated *in vivo*. In our patient, the absence of fever or rash and the coexistence of vasoplegic, cardiogenic and obstructive shock physiology delayed recognition and complicated management. Despite early antitoxin antibiotics, systemic thrombolysis for high-risk pulmonary embolism and salvage VA-ECMO, the outcome was fatal. Greater awareness of such atypical, highly lethal presentations is needed to support earlier recognition and refine intensive care strategies.

## Data Availability

The original contributions presented in the study are included in the article/supplementary material, further inquiries can be directed to the corresponding author.
